# Adequacy of diabetes care for older U.S. rural adults: a cross-sectional population based study using 2009 BRFSS data

**DOI:** 10.1186/1471-2458-11-940

**Published:** 2011-12-16

**Authors:** M Nawal Lutfiyya, Joel E McCullough, Lori Mitchell, L Scott Dean, Martin S Lipsky

**Affiliations:** 1 Essentia Institute of Rural Health, Research Division, Duluth, MN, 55805, USA; 2 Spokane Regional Health District, 1101 West College Avenue, Spokane, WA 99201, USA; 3 Winnipeg Regional Health Authority, Home Care Program, 650 Main Street, Winnipeg, Manitoba, R3B 1E2, Canada; 4 Charleston Area Medical Center, Health Education and Research Institute, 3211 MacCorkle Ave, Charleston, WV 25314, USA; 5 Department of Community and Family Medicine, University of Illinois College of Medicine at Rockford, 1601 Parkview Avenue, Rockford, Il 61107, USA

## Abstract

**Background:**

In the U.S. diabetes prevalence estimates for adults ≥ 65 years exceed 20%. Rural communities have higher proportions of older individuals and health disparities associated with rural residency place rural communities at risk for a higher burden from diabetes. This study examined the adequacy of care received by older rural adults for their diabetes to determine if older rural adults differed in the receipt of adequate diabetes care when compared to their non-rural counterparts.

**Methods:**

Cross-sectional data from the 2009 Behavioral Risk Factor Surveillance Survey were examined using bivariate and multivariate analytical techniques.

**Results:**

Logistic regression analysis revealed that older rural adults with diabetes were more likely to receive less than adequate care when compared to their non-rural counterparts (OR = 1.465, 95% CI: 1.454-1.475). Older rural adults receiving less than adequate care for their diabetes were more likely to be: male, non-Caucasian, less educated, unmarried, economically poorer, inactive, a smoker. They were also more likely to: have deferred medical care because of cost, not have a personal health care provider, and not have had a routine medical check-up within the last 12 months.

**Conclusion:**

There are gaps between what is recommended for diabetes management and the management that older individuals receive. Older adults with diabetes living in rural communities are at greater risk for less than adequate care when compared to their non-rural counterparts. These results suggest the need to develop strategies to improve diabetes care for older adults with diabetes and to target those at highest risk.

## Background

Diabetes is a chronic metabolic condition characterized by elevated blood glucose levels related to insulin resistance and impaired insulin secretion [1,2]. Type 2 diabetes accounts for more than 95% of diabetes cases in the United States and affects approximately 8% of the adult population [1-5]. Diabetes can cause microvasculcar disease affecting the eyes, kidneys and nerves, [2,4] and increases the risk for peripheral vascular disease, heart disease and stroke. Diabetes is currently the sixth leading cause of death in the United States [1] and older adults with diabetes experience twice the mortality of age-matched controls without diabetes [6].

In 2007, the American Diabetes Association (ADA) estimated the total economic cost of diabetes at $174 billion [7]. This dollar amount included both direct and indirect costs and represented a 32% increase for total diabetes-related costs from 2002 cost estimates [7]. The ADA cited increased growth in diabetes prevalence as a major cause for the raising costs of diabetes in the United States [7]. While diabetes affects all age groups, studies consistently demonstrate that the prevalence of type 2 diabetes is strongly associated with increased age [8-12]. Using data collected from 2003-2007, Danaei, et al., estimated that the overall prevalence for diagnosed diabetes for U.S. adults ≥ 30-59 years was 8.4% while for adults ≥ 60 years it was 23.8% [13]. As is the case with a number of chronic conditions, the burden of diabetes is not shared evenly across all racial and ethnic groups [1,13-16]. In the U.S., Hispanic adults and African American adults experience disproportionately higher rates of diabetes. For adults over age 65, the diagnosed diabetes prevalence for U.S. Hispanic adults was 27.8% and 29.7% for African American [17].

Primary prevention for type 2 diabetes depends on managing modifiable risk factors such as elevated BMI, nutrition and physical inactivity;[4,5,9,11,18,19] while secondary prevention entails screening for disease before symptoms of the disease are present [20] and tertiary prevention requires both managing modifiable risk factors and adequacy of care [21-25]. Adequacy of diabetes care requires that individuals with diabetes receive care consistent with accepted clinical practice guidelines [3-5,11,12]. Optimizing lipid levels and blood pressure, conducting dilated eye examinations, monitoring retinopathy and checking feet to monitor for neuropathy are examples of recommended components of diabetes management [3-5,11,21,26,27]. In addition, formal diabetes education is a key element of optimal care [24,25,28].

As an age prevalent disease, the prevalence and impact of diabetes will likely continue to increase as the population in the U.S. ages. The U.S. Census Bureau estimates that the populations of those over age 65 will more than double by 2050, [29] highlighting the importance of optimizing diabetes management in older individuals. Since rural populations have a larger proportion of older adults compared to non-rural areas, [30] these communities are potentially more vulnerable to the burden of diabetes.

While rural and non-rural areas differ in many ways such as in their environment and demography, only recently have public health researchers recognized that rural residency may be associated with disparities in health and health care [30]. Lutfiyya, et al., examined differences in diabetes care between U.S. rural and non-rural adults and found that rural adults were more likely to receive inadequate care for their diabetes when compared to their non-rural counterparts [21]. However, they did not separately evaluate the care of older adults. Since the majority of adults over age 65 in the US have Medicare insurance this may impact the level of diabetes care received in older adults compared to younger individuals. Moreover, several recent publications suggest the need for more focus on chronic disease care guidelines for older adults [9,12,16]. Likewise, other epidemiological research on older adults with diabetes has not considered rural residency as a separate and potentially at risk population group.

In this study we sought to fill in some important gaps in the epidemiological literature by examining the adequacy of care received by adults 65 years of age and older. By using a dependent variable *less than adequate diabetes care *computed from eleven other variables, this study also explored whether there were differences in adequacy of diabetes care between older rural adults when compared to their non-rural counterparts.

## Methods

To answer the research question, data from the 2009 Behavioral Risk Factor Surveillance Survey (BRFSS) were examined. The population of interest was older (≥ 65 years) U.S. rural and non-rural adults with diabetes. The study used BRFSS data from the 2009 survey because for that year 38 states or territories chose to use the optional adult diabetes module to generate surveillance data. While more states used the diabetes module in the subsequent year of available data, other survey questions deemed important (i.e., questions regarding blood pressure and cholesterol testing) were not asked. Data generated from questions from both the core BRFSS survey as well as the adult BRFSS diabetes module were used in the analyses conducted for this research study.

BRFSS is a cross-sectional, random digit telephone survey that is a collaborative project of the Centers for Disease Control and Prevention (CDC) and all U.S. states and territories. The survey measures several behavioral risk factors and disease states in the non-institutionalized adult population aged 18 through 97 years. Data are collected from a random sample of adults (one per household). A more detailed description of the sampling methodology of BRFSS is available elsewhere [31]. All BRFSS data are self-reported responses to mostly forced-choice questions. As recommended by the CDC, all analyses were performed on weighted data. The weighting provides a stratified representation of the U.S. adult non-institutionalized population and conforms to census data patterns.

For analysis purposes, a number of variables from the dataset were either re-coded or computed. The Metropolitan Statistical Area (MSA) variable included in BRFSS was used to define place of residence and was re-coded into the dichotomous categories of rural and non-rural. Rural residents were defined as people living either within an MSA that had no city center or outside an MSA. Non-rural residents included all respondents living in a center city of an MSA, outside the center city of an MSA but inside the county containing the center city, or inside a suburban county of the MSA.

Adequacy of diabetes care constituted the dependent variable for this study. This was a computed variable derived from responses given to eleven questions and entailed both self-care as well as care received from a health care provider. The answers given by respondents to each of these eleven questions were collapsed into bifurcated categories. For our analyses, we created a diabetes care index from the following eleven bifurcated variables: 1) self-glucose test at least daily, 2) self-foot check at least daily, 3) have controlled blood pressure, 4) had cholesterol checked in past 12 months, 5) had flu vaccination in past 12 months, 6) had lifetime pneumonia vaccination, 7) had 2 diabetes checks in past 12 months, 8) health care provider checked A1c twice in past 12 months, 9) health care provider checked feet twice in past 12 months, 10) had dilated eye exam in past 12 months, and 11) have had lifetime diabetes education. These variables were chosen because they reflected clinical practice recommendations for older adults with diabetes [5,9-11]. Respondents were excluded from the analysis if data were missing on any aspect of the adequacy variable.

Optimal care was defined as getting all 11 of the care interventions. Since no single practice recommendation includes all 11 variables, the authors believed that providers who might not do all 11 interventions could still be considered as providing adequate care. After deliberation the consensus among the authors was to define "adequate care" as adults with diabetes who received at least nine of the 11 interventions. This allowed for instances where providers might have legitimate reasons for not complying with all the interventions yet could still be considered as providing adequate care for their older patients with diabetes. Face validity for this definition as a meaningful measure to compare care was confirmed through feedback from colleagues of the authors caring for older individuals with diabetes. Less than adequate care was defined as getting eight or fewer of these 11 care interventions.

Race and ethnicity was a computed variable calculated from participant responses to two separate survey questions---one regarding race and the other regarding Latino/Hispanic ethnicity. Combining the responses to these two questions allowed for the derivation of the race and ethnicity variable used in the analyses presented here. All race/ethnicity categories were computed as mutually exclusive entities. For example all respondents coded as Caucasian chose White as their racial classification, likewise black for African American, etc. If a respondent identified themselves as Hispanic, they were classified by that ethnic category regardless of any additional racial classification. The category of other/Multiracial was also calculated. All racial categories were non-Hispanic.

Controlled blood pressure was computed from two separate variables: *high blood pressure *(yes/no) and *taking blood pressure medication for high blood pressure *(yes/no). Respondents reporting that they did not have high blood pressure as well as those reporting that they had high blood pressure and were taking medication to control it were coded as having controlled blood pressure. Respondents reporting that they had high blood pressure but were not taking medication for it were coded as having uncontrolled blood pressure.

Level of physical activity, another covariate, was also computed by combining two other variables assessing physical activity level: 1) whether or not a person was getting recommended levels of moderate physical activity, and 2) whether or not a person was getting recommended levels of vigorous physical activity. People who reported getting recommended levels of either moderate or vigorous physical activity were coded as getting at least recommended levels of moderate physical activity. Recommended levels of moderate physical activity were defined as: moderate-intensity activities such as brisk walking for at least 30 minutes per day, at least 5 days per week.

For this research study the covariates where response categories were collapsed into the fewer categories were:

Education (Did Not Graduate From High School/Graduated From HS/College Graduate)

Annual Household Income (< $35,000/> = 35,000)

Marital Status (Married or Living with Partner/Unmarried and Not Living With a Partner)

Self-Reported Health Status (Fair To Poor Health/Good To Excellent Health)

Have Own Health Care Provider (Yes/No)

BMI (BMI < 25/BMI 25- < 30/BMI > = 30)

Smoking Status (Yes/No)

Timing of Last Routine Medical Check-up **(**Within the Past 12 Months/More than 12 Months Ago)

Diabetes (Yes/No)

Insulin Use (Yes/No)

Diabetes Has Affected Eyes (Yes/No)

One continuous variable, age of diabetes onset, was recoded into a categorical variable with three levels: < 45 years, 45-64 years, and ≥ 65 years. Three additional variables: sex, have health insurance, and medical care deferment because of cost, were also included in the analyses as covariates. The only re-coding efforts involved with these last three variables entailed the removal of missing data.

Bivariate analysis using contingency tables was conducted to determine whether or not there were differences between rural and non-rural older adults with diabetes in terms of demographic and health services characteristics as well as diabetes care variables.

Two multivariate logistic regression models were performed to characterize older U.S. adults with diabetes receiving less than adequate care. In the logistic regression analysis those with missing data on the covariates were excluded. Less than adequate diabetes care was the dependent variable for the 2 separate logistic regression models---one that included all older U.S. adults with diabetes, one including only older rural adults with diabetes.

For all statistical analyses, alpha was set at p < 0.05. Statistical Package for Social Scientists (SPSS, IBM, Chicago, IL) version 19.0 was used to complete all statistical analyses performed for this study. Human subject approval was sought and received from Essentia Health's IRB as well as the University of Illinois, College of Medicine at Rockford's IRB.

## Results

The weighted data analyzed included survey information on 39,942,447 older U.S. adults, 7,946,892 of whom reported that they had been diagnosed with diabetes leading to an overall diabetes prevalence of 20.2% for U.S. adults aged 65 and older. These data were weighted from 133,305 respondents 65 years and older where 25,415 reported that they had been diagnosed with diabetes. Of these older U.S. adults reporting that they had a diabetes diagnosis, 16,145 were non-rural residents and 9,270 rural residents.

Our analysis indicated that the burden of diabetes was unequally shared by different sub-groups of older adults. Over one-third (34.7%) of older U.S. African American adults reported being diagnosed with diabetes while the older adult Hispanic prevalence for diabetes was 30.1%. Almost a quarter of older adults living in households with annual incomes < $35,000 reported having been diagnosed with diabetes (24.3% prevalence) while the prevalence of diabetes for older adults with < a high school education was 28.76%.

Table 1 displays the bivariate analysis comparing older U.S. adults with diabetes by place of residency (non-rural and rural) and by demographic and health service variables. This analysis yielded that the rural older adult population with diabetes in contrast to the non-rural older adult population with diabetes had a higher proportion of: Caucasians than non-Caucasians (81.2% vs. 67.0%), those with less than a high school education (23.2% vs. 18.9%), those living in households with annual incomes less than $35,000 (71.0% vs. 61.5%), those not having had a routine medical check-up within the past 12 months (11.4% vs. 8.8%), and those self-defining their health as fair to poor (49.0% vs. 43.4%).

**Table 1:** Demographic and Health Service Characteristics of Older U.S. Adults with Diabetes by Geographic Locale (Rural or Non-Rural)* 2009 BRFSS Data (weighted n = 7946892)

Variables and Factors		% Non-Rural (weighted n = 6191312)	% Rural (weighted n = 1755580)
Demographic Variables			

Sex	Male	47.5	48.3
	
	Female	52.5	51.7

Race/Ethnicity	Caucasian	67.0	81.2
	
	African American	15.1	8.1
	
	Hispanic	11.0	4.1
	
	Other/Multiracial	7.0	6.6

Education	< High School	18.9	23.2
	
	Completed High School	58.1	62.2
	
	College Graduate	23.0	14.7

Marital Status	Married Or Living With Partner	56.6	60.3
	
	Unmarried And Not Living With A Partner	43.4	39.7

Household Income	< $35,000	61.5	71.0
	
	> = $35,000	38.5	29.0

Health Service Variables			

Have Health Insurance	Yes	98.0	97.6
	
	No	2.0	2.4

Deferment of Medical Care Because of Cost	Deferred Medical Care Because of Cost	6.0	5.7
	
	Did Not Defer Medical Care Because of Cost	94.0	94.3

Have A Health Care Provider	Yes	96.7	96.0
	
	No	3.3	4.0

Timing of Last Routine Medical Check-up	Within Last 12 Months	91.2	88.6
	
	Longer Than 12 Months Ago	8.8	11.4

Self-Defined Health Status	Good To Excellent	56.6	51.0
	
	Fair To Poor	43.4	49.0

* In all instances column proportions differ significantly from each other at the .05 level.

Table 2 compares the health care characteristics of older U.S. adults with diabetes by geographic locale. This bivariate analysis revealed that a higher proportion of the older rural adults with diabetes: use insulin (27.7% vs. 25.3%), have a BMI ≥ 30 (42.8% vs. 40.7%), have not had their cholesterol checked within the past 12 months (11.5% vs. 8.6%), have not had diabetes education (69.1% vs. 64.1%), and have received less than adequate diabetes care (76.9% vs. 74.8%). The differences in optimal care were small with 4.7% of older rural adults receiving all 11 interventions compared with 5.1% of older non-rural adults with diabetes.

**Table 2:** Diabetes Health Care Characteristics of Older U.S. Adults with Diabetes by Geographic Locale (Rural or Non-Rural)* 2009 BRFSS Data (weighted n = 7946892)

Variables and Factors		% Non-Rural (weighted n = 6191312)	% Rural (weighted n = 1755580)
Age of Diabetes Onset	< 45 Years	10.4	10.4
	
	45 - 64 Years	47.6	47.7
	
	> = 65 Years	42.0	41.9

Insulin Use	Yes	25.3	27.7
	
	No	74.7	72.3

BMI	BMI < 25	21.3	18.0
	
	BMI 25- < 30	38.0	39.2
	
	BMI ≥ 30	40.7	42.8

Physical Activity	Getting At Least Moderate Physical Activity	31.2	31.3
	
	Inactive	68.8	68.7

Smoking Status	Smoker	13.5	14.2
	
	Non-Smoker	86.5	85.8

Diabetes Has Affected Eyes	Yes	19.2	20.5
	
	No	80.8	79.5

Self-Glucose Check	< Daily	57.3	54.7
	
	Daily Check	42.7	45.3

Self-Foot Check	< Daily	58.2	55.0
	
	Daily Check	41.8	45.0

High Blood Pressure (HBP)	Do Not Have HBP	25.5	24.6
	
	Have HBP	74.5	75.4

Controlled Blood Pressure	Controlled	97.2	96.8
	
	Not Controlled	2.8	3.2

Last Cholesterol Check	> 12 Months Ago	8.6	11.5
	
	Within Last 12 Months	91.4	88.5

Seasonal Flu Vaccination	No	30.0	29.5
	
	Yes	70.0	70.5

Lifetime Pneumonia Vaccination	No	33.9	27.8
	
	Yes	66.1	72.2

Lifetime Diabetes Education	No	64.1	69.1
	
	Yes	35.9	30.9

Diabetes Check-up	< Twice In Past 12 Months	50.5	50.3
	
	At least Twice In Past 12 Months	49.5	49.7

Health Care Provider Checked A1c	< Twice In Past 12 Months	57.9	58.1
	
	At least Twice In Past 12 Months	42.1	41.9

Health Care Provider Checked Feet	< Twice In Past 12 Months	63.1	64.7
	
	At least Twice In Past 12 Months	36.9	35.3

Dilated Eye Exam	Longer Than 12 Months Ago	47.4	50.0
	
	Within Last 12 Months	52.6	50.0

Adequacy of Diabetes Care	Adequate Care	25.2	23.1
	
	Less Than Adequate Care	74.8	76.9

*In all instances column proportions differ significantly from each other at the .05 level.

Two multivariate logistic regression models were performed using *less than adequate diabetes care *as the dependent variable and the results of this analysis are displayed in Table 3. The first model included all older U.S. adults with diabetes, the second included only rural adults. Sixteen categorical covariates were entered into the first model, and 15 into the second (geographic place of residency was removed from the second model). Only one covariate---health insurance---did not demonstrate a statistically significant relationship in both models. Both models were robust, explaining over 80% of the cases of interest.

**Table 3:** Multivariate Logistic Regression Models: Characteristics of Older U.S Adults and Older Rural Adults with Diabetes Receiving Less Than Adequate Care for Diabetes 2009 BRFSS Data

Variables and Factors		Adjusted Odds Ratios (95% CI)	
		
		All Older U.S. Adults	Older Rural U.S. Adults
Sex	Male	1.638 (1.627, 1.648)	1.547 (1.525,1.569)
	
	Female	---*	---*

Race/Ethnicity	Caucasian	---*	---*
	
	African American	1.084 (1.074, 1.094)	3.103 (3.009, 3.200)
	
	Hispanic	1.503 (1.480, 1.525)	2.138 (2.062, 2.217)
	
	Other/Multiracial	1.174 (1.158, 1.192)	1.120 (1.086, 1.154)

Education	< High School	1.793 (1.774, 1.812)	1.565 (1.529, 1.602)
	
	Completed High School	1.274 (1.265, 1.283)	1.403 (1.377, 1.429)
	
	College Graduate	---*	---*

Marital Status	Married or Living With Partner	---*	---*
	
	Unmarried and not Living With a Partner	1.258 (1.249, 1.266)	1.096 (1.080, 1.112)

Household Income	< $35,000	1.225 (1.216, 1.233)	1.061 (1.044, 1.077)
	
	> = $35,000	---*	---*

Geographic Locale	Non-Rural	---*	---*
	
	Rural	1.465 (1.454, 1.475)	n/a

Self-Defined Health Status	Good to Excellent	---*	---*
	
	Fair to Poor	.720 (.715, .724)	.601 (.593, .610)

Age Diabetes Onset	< 45 Years	---*	---*
	
	45 - 64 Years	1.077 (1.066, 1.089)	1.087 (1.061, 1.113)
	
	> = 65 Years	1.321 (1.306, 1.336)	1.022 (.997, 1.048)*

Insulin Use	Yes	.422 (.419, .425)	.494 (.487, .502)
	
	No	---*	---*

BMI	BMI < 25	---*	---*
	
	BMI 25- < 30	.983 (.975, .991)	.961 (.942, .979)
	
	BMI > = 30	.845 (.838, .852)	.973 (.955, .991)

Physical Activity	Getting at Least Moderate Physical Activity	---*	---*
	
	Inactive	1.169 (1.162, 1.176)	1.363 (1.344, 1.382)

Smoking Status	Smoker	1.620 (1.606, 1.634)	1.471 (1.442, 1.500)
	
	Non-Smoker	---*	---*

Have Health Insurance	Yes	---*	---*
	
	No	.991 (.967, 1.015)	1.027 (.984, 1.072)

Deferment of Medical Care Because of Cost	Deferred Medical Care Because of Cost	1.717 (1.694, 1.740)	2.871 (2.775, 2.971)
	
	Did not Defer Medical Care Because of Cost	---*	---*

Have a Health Care Provider	Yes	---*	---*
	
	No	1.453 (1.426, 1.481)	1.093 (1.055, 1.132)

Timing of Last Routine Medical Check-up	Within Last 12 Months	---*	---*
	
	Longer Than 12 Months Ago	2.136 (2.112, 2.160)	2.453 .390, 2.516)

---* Reference Category

The results from the first logistic regression model yielded that older U.S. adults with diabetes receiving less than adequate care for their diabetes were more likely to be rural rather than non-rural residents (OR = 1.465 95% CI 1.454-1.475). The results from the second logistics regression model that included only older U.S. rural adults with diabetes yielded that those receiving less than adequate care for their diabetes were more likely to: be male (OR = 1.547 95% CI 1.525-1.569) than female; be African American (OR = 3.103 95% CI 3.009-3.200), Hispanic (OR = 2.138 95% CI 2.062-2.217), or other/Multiracial (OR = 1.120 95% CI 1.086-1.154) than Caucasian; have less than a university education (< HS, OR = 1.565 95% CI 1.529-1.602, HS graduate OR = 1.403 95% CI 1.377-1.429); be unmarried and/or not living with a partner (OR = 1.096 95% CI 1.080-1.112); have a household income < $35,000 (OR = 1.061 94% CI 1.044-1.077); be physically inactive (OR = 1.363 95% CI 1.344-1.382) rather than getting at least moderate levels of physical activity; and be a smoker (OR = 1.471 95% CI 1.442-1.500) rather than a non-smoker. In addition older rural adults with diabetes receiving less than adequate care were also more likely to: have deferred medical care because of cost (OR = 2.871 95% CI 2.775-2.971); not have had a healthcare provider (OR = 1.093 95% CI 1.055-1.132); and to have had their last routine check-up longer than 12 months ago (OR = 2.453 95% CI 2.390-2.516). Finally, older rural adults with diabetes receiving less than adequate care were less likely to use insulin (OR = .494 95% CI .487-.502) or have an elevated BMI.

## Discussion

Diabetes is highly prevalent and increased in persons aged 65 and older. Similar to previous studies [17] this study found that overall about one in five individuals over age 65 has diabetes, a prevalence rate that has doubled over the past decade [32]. Analogous to the epidemiology of younger populations with diabetes, we found that in older individuals minority groups were disproportionately affected with African Americans and Hispanics experiencing substantially higher prevalence rates than Caucasians. Older adults with diabetes report higher rates of functional disability than those without diabetes [33-35] and consistent with this our findings revealed that the percentage of adults with diabetes who rated their health as fair to poor (45.2%) was twice that of those without diabetes (22.1%). These findings support the concept that diabetes is an emerging epidemic with serious economic, social and health related implications for older Americans [36-38].

In general, overall management strategies for diabetes in older adults are similar to those for younger individuals [37,38]. Once a person develops diabetes, adequate care can delay or reduce the impact of the complications associated with diabetes. Despite the progress in diabetes care, not all individuals receive the care recommended by clinical guidelines [5,9-11]. Our findings indicated that about three in four older adults received less than adequate care and only 4.7% of older rural adults and 5.1% of older non-rural adults with diabetes received optimal care. While disparities in rural settings are often viewed as a proxy for access to care and access to specialists for care, after controlling for variables such as health insurance, having a health care provider, income, and education rural residents were still 45% more likely to receive *less adequate care*.

Although previous research indicates that rural adults experience disparities in diabetes care, [21] this is the first study using a nationally representative sample of *older *adults that documents a difference in diabetes care for older *rural *adults. Our findings support the concept that there is a gap in diabetes care between rural and non-rural patients [21] and the gap is even greater for older individuals with diabetes. The findings reported here also add to a growing body of literature about the impact of geographic locale on health and rural residency as an independent marker of health disparity [39]. Rural residents at greater risk for less than adequate diabetes care were: males, non-Caucasians, and those less educated, poorer, unmarried or living without a partner [13,17]. Previous research also associates disparities in diabetes management with sociodemographic characteristics such income level, educational attainment, health insurance, and racial/ethnic background.

Explanations for health disparities should include both individual characteristics (people factors) and the social environment (social structural factors) [40] since people are simultaneously social and biological entities [41]. Type 2 diabetes is influenced by both micro (individual) and macro (societal) factors [40]. We hypothesize that in addition to distinct individual patterns of disparities, diabetes management is influenced by factors such as the prevalence of food deserts [42-45] and environments built to disfavor physical activity [42,46-48] that reflect the places where rural residents and older, low income, and often minority populations live [42,43,48]. These factors create not only a greater vulnerability to ill health but likely contribute to the patterns of diabetes prevalence and care.

Furthermore, while access to medical care facilities and specialist providers have long been recognized as factors influencing the health of rural residents, focusing on these issues alone will not eliminate the disparities surrounding diabetes and diabetes care. Our findings suggest that to be successful, intervention strategies to improve outcomes will need to address the social and geographic context for individuals with chronic disease [38], and the evident deferment of medical care because of cost that can put these older adults at greater risk.

## Limitations

Several potential limitations to this study should be noted. First, the survey is based on telephone derived data and may be skewed because those who could not be reached by phone could not participate in the survey. Wide-spread use of answering machines and caller ID allow people to filter their phone calls potentially leading to a passive refusal to participate in surveys such as the BRFSS. However, call filtering is beyond the control of survey administrators. In addition, persons of lower socioeconomic status may have been excluded because of poorer phone access, but the vast majority of U.S. residents live in households with telephones, which minimizes this bias. Furthermore, U.S. cell phone numbers are now included in the pool of phones contacted for the survey. In addition, study strength is in the use of a national database that included a robust sample of rural residents weighted to reflect the demographics of rural vs. non-rural U.S. populations.

A second limitation is that the survey used close-ended questions, which limit a responder's options to fully explain response choices. Nonetheless, the survey questions were worded such that the answer choices covered a wide range of response possibilities. A third and related limitation is that the answers are self-reported, which introduces the possibility of recall bias on the part of the survey participants.

Fourth, only those variables available from the survey questions could be used and these questions may not reflect a fully comprehensive measure of diabetes care. For instance, having a cholesterol check may not mean a provider appropriately managed an abnormal level. However this is no reason to suspect a systematic bias between rural and non-rural providers suggesting the definition of adequacy of care used in the analysis should allow for meaningful comparisons.

A fifth potential bias resulted from the languages of the survey - English and Spanish. Individuals who did not speak English or Spanish were excluded from this survey. Finally we recognize that our definition for less than adequate diabetes care might be challenged. However the same definition was applied to all groups analyzed, thus allowing for meaningful comparisons.

## Conclusions

There are still gaps between what is recommended for diabetes management and the management that older individuals receive. Those living in rural communities are at greater risk for less than adequate care when compared to their non-rural counterparts. These findings suggest the need to develop strategies to improve care in older adults and to target those at highest risk. Primary care providers caring for older adults with diabetes, especially those providing care for older rural adults with diabetes, should use the findings of this study to examine the care they are currently providing and implement adjustments as necessary.

## Competing interests

The authors declare that they have no competing interests.

## Authors' contributions

MNL, JEM, LM, LSD, MSL all made substantial contributions to conception and design of the manuscript, contributed to the interpretation of the data, were involved in revising the manuscript critically for important intellectual content, and have given final approval of this version of the manuscript to be published. Additionally, MNL completed: the first draft of the manuscript, the acquisition of the data and the statistical analyses. All authors read and approved the final version of this manuscript.

## Pre-publication history

The pre-publication history for this paper can be accessed here:

http://www.biomedcentral.com/1471-2458/11/940/prepub
